# Targeting obesity-related dysfunction in hormonally driven cancers

**DOI:** 10.1038/s41416-021-01393-y

**Published:** 2021-04-28

**Authors:** Maria M. Rubinstein, Kristy A. Brown, Neil M. Iyengar

**Affiliations:** 1 Department of Medicine, Memorial Sloan Kettering Cancer Center, New York, NY USA; 2 Department of Biochemistry in Medicine, Weill Cornell Medical College, New York, NY USA

**Keywords:** Cancer microenvironment, Oncogenesis, Cancer metabolism, Cancer therapy

## Abstract

Obesity is a risk factor for at least 13 different types of cancer, many of which are hormonally driven, and is associated with increased cancer incidence and morbidity. Adult obesity rates are steadily increasing and a subsequent increase in cancer burden is anticipated. Obesity-related dysfunction can contribute to cancer pathogenesis and treatment resistance through various mechanisms, including those mediated by insulin, leptin, adipokine, and aromatase signalling pathways, particularly in women. Furthermore, adiposity-related changes can influence tumour vascularity and inflammation in the tumour microenvironment, which can support tumour development and growth. Trials investigating non-pharmacological approaches to target the mechanisms driving obesity-mediated cancer pathogenesis are emerging and are necessary to better appreciate the interplay between malignancy, adiposity, diet and exercise. Diet, exercise and bariatric surgery are potential strategies to reverse the cancer-promoting effects of obesity; trials of these interventions should be conducted in a scientifically rigorous manner with dose escalation and appropriate selection of tumour phenotypes and have cancer-related clinical and mechanistic endpoints. We are only beginning to understand the mechanisms by which obesity effects cell signalling and systemic factors that contribute to oncogenesis. As the rates of obesity and cancer increase, we must promote the development of non-pharmacological lifestyle trials for the treatment and prevention of malignancy.

## Background

The rates of adult obesity are increasing yearly and have already reached epidemic proportions.^1^ Obesity—classically defined as a body mass index (BMI) of 30 or greater^2^—is a well-known contributor to overall mortality and, specifically, to death from cardiometabolic diseases like diabetes and coronary artery disease.^3^ In the past decade, the links between obesity and rising rates of cancer incidence and cancer-specific death have been increasingly recognised, and current evidence implicates obesity as a risk factor for at least 13 different types of cancer, including oesophageal, gastric, colorectal, breast and endometrial cancers.^4,5^ The relative risks of oesophageal, gastric and colon cancer for obese individuals are 4.8-, 1.8-, and 1.3-fold greater, respectively, and a staggering 7.1-fold greater for endometrial cancer, than those for non-obese individuals.^5^ A longer exposure time to obesity is also associated with an increased incidence of many of these cancers.^5,6^ As the number of young obese and overweight individuals continues to rise, a related acceleration in the global cancer burden is likely to follow. Indeed, this prediction has already been realised by the increased incidence of endometrial, gallbladder, pancreatic and other obesity-related cancers in younger cohorts (25–29 years old).^7^ In terms of mortality, it is estimated that elevated body weight and excess adiposity negatively impact clinical outcomes in ~20% of all cancer cases.^8^ In obese women, observational studies indicate a 2.12-fold increase in the relative risk of death from breast cancer and a 6.25-fold increase in the relative risk of death from uterine cancer.^9^ In men, obesity has been associated with more than quadruple and nearly double the risk of death from liver and colorectal cancers, respectively.^9^

The recognition of obesity as a leading modifiable risk factor for cancer development and mortality has triggered an active area of investigation and a rationale for testing anti-obesity interventions in oncology. Weight loss strategies targeting overweight or obese individuals account for most of these interventions. Despite multiple completed and ongoing clinical trials, however, it is still unclear whether weight loss reduces the risk of developing cancer and/or cancer-related death.^10^ Reliance on diagnostics that are useful for the assessment of population health but imprecise at the individual level might, in part, contribute to the challenge of identifying successful interventions for obesity-related cancers. Indeed, nearly all large epidemiology studies use convenient but imprecise surrogates of adiposity (e.g., BMI, waist circumference) to approximate the impact of obesity on cancer. However, such anthropometric measures frequently mischaracterise obesity-related dysfunction and related disease incidence. For example, nearly one-third of women with normal BMI (<25 kg/m^2^) have subclinical evidence of metabolic obesity.^11^ Similarly, increased adiposity is associated with a two-fold increase in the risk of invasive breast cancer among postmenopausal women with normal BMI.^12^ Conversely, up to 30% of obese individuals can be defined as metabolically healthy.^13^ The developmental paradigm of interventions for obesity-related cancers must therefore incorporate a more precise characterisation of disease phenotype in order to parallel the successes of other cancer therapies that target specific biological pathways (Box 1).

The scope of obesity-related malignancy is vast and varied. In this review, we will discuss the need to characterise obesity via biological targets that are relevant to oncological pathways to facilitate mechanistically driven and precise interventions for obesity-related cancers. We will focus on hormonally driven cancer, such as breast and endometrial cancers, and on the changes in peptide and steroid hormones, including insulin and insulin-like growth factors (IGFs), various adipokines such as leptin and adiponectin, and oestrogen, that link metabolic dysfunction with chronic low-level systemic inflammation. Finally, we will discuss the translation of biological findings into the development of interventions, with a focus on lifestyle modification strategies, that aim to attenuate the drivers of obesity-induced tumorigenesis.

Box 1 White adipose tissue (WAT)

- Predominant type of fat
- Anatomical locations include:
  - Subcutaneous
  - Visceral
  - Bone marrow
  - Breast
- Most common solid tumours arise within organs containing or surrounded by WAT

## Obesity and dysregulated insulin signalling

**Fig. 1:**
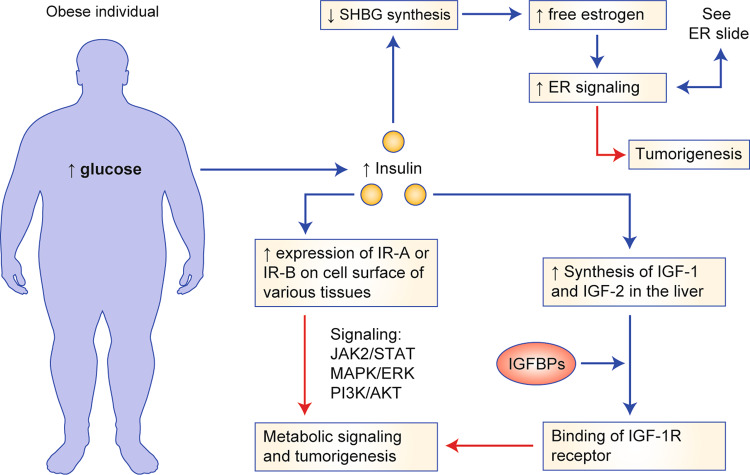
Affects of Obesity on Insulin and Estrogen Signaling. IR-A Insulin Receptor A, IR-B Insulin Receptor B, IGF-1 Insulin Growth Factor-1, IGF-2 Insulin Growth Factor 2, IGF-1R Insulin Growth Factor Receptor 1, SHBG Sex Hormone Binding Globulin, ER Estrogen receptor.

Under normal physiological conditions, increases in the levels of systemic glucose induce pancreatic cells to release the hormone insulin, which, in addition to mediating glucose metabolism, stimulates key pathways implicated in cell survival, protein synthesis and replication.^14^ It does this by binding to insulin receptors (IR) on the surface of cells and activating various signalling cascades, including the extracellular-signal-regulated kinase (ERK)/mitogen-activated protein kinase (MAPK) and the phosphatidylinositol 3-kinase (PI3K) pathways^15–17^ (Fig. 1). Two IR isoforms, IR-A and IR-B, are present among various tissues in different ratios and carry out different functions. IR-A has largely a mitogenic role in early life,^18^ and its expression in adulthood is linked to insulin resistance and unregulated cell proliferation.^19^ In comparison, IR-B is expressed in the liver and other differentiated adult tissue and is involved primarily in glucose metabolism.^18^ High levels of IR-A are implicated in tumorigenesis and are found in various cancers including breast, endometrial, colon and hepatocellular cancer.^20–22^ IR-A also shows an increased affinity for IGFs compared with IR-B.^23^ IGF-1 and IGF-2 are small peptides synthesised in the liver in response to growth hormone. While insulin circulates mostly in its free form, IGFs circulate largely bound to IGF-binding proteins (IGFBPs), which regulate their levels and biological function.^24^ By binding to an IGF-1 receptor (IGF-1R), IGF-1 and IGF-2 promote cell growth and proliferation,^25^ and several studies have demonstrated that the expression of IGF-1R is increased in breast and endometrial cancers.^21,26,27^ In the setting of obesity, higher levels of IGF-2 stimulate both IGF-1R and IR-A.^28^

Although genetic mutation of IGF-1R as the primary driver event in tumorigenesis is infrequent, dysregulation of the IGFR axis can occur secondary to other events that influence the expression of ligands and receptors in this pathway.^29–32^ Several factors allow transformed malignant cells to heavily depend on the dysregulation of insulin and IGF signalling pathways for proliferation and invasion.^32^ In a state of energy abundance, such as occurs in obesity, insulin and IGFRs are chronically activated, resulting in increased glucose uptake into cells, cell proliferation, angiogenesis and, ultimately, greater potential for malignant transformation and growth.^33,34^ Additionally, hyperinsulinaemia and insulin resistance occur in the setting of excess visceral adiposity,^35^ but are not strongly associated with subcutaneous or total body adiposity;^36^ this difference is thought to be mediated by a rise in circulating free fatty acids due to increased rates of lipolysis in visceral, but not subcutaneous, fat depots.^37^ Although the mechanisms through which excess free fatty acids released from visceral adipose tissue cause insulin resistance remain the subject of ongoing investigation, proposed mechanisms include production of lipid metabolites and secretion of pro-inflammatory cytokines that stimulate insulin release.^38^ Accordingly, in studies that differentiate adipose compartments, visceral adiposity is associated with an increased risk of several cancers and is a stronger predictor of risk than BMI.^37^ For breast cancer, specifically, elevated insulin levels in non-diabetic patients are associated with worse progression-free survival (PFS) then normal insulin levels, and patients with obesity and diabetes have significantly higher mortality rates compared with non-diabetics.^9,39^ Hyperactivation of IR and IGFR also promotes downstream signalling through PI3K, which is dysregulated and constitutively activated in various obesity-associated cancers, including breast, endometrial and colorectal cancers.^40–42^ Inhibition of PI3K is a strategy that is currently used in cancer treatment and is associated with on-target hyperglycaemia and hyperinsulinaemia.^43^ In preclinical models, the subsequent surge in insulin after PI3K inhibition can reactivate this pathway and stimulate further tumour-cell proliferation.^44^ Thus, insulin resistance, characterised by prolonged periods of hyperinsulinaemia and stimulation of IR-A and IGFR1, is a key means by which obesity promotes the development and growth of cancer.

### Targeting the insulin signalling pathway

Strategies that target insulin and IGF signalling for cancer treatment include ligand- or receptor-specific agents, as well as interventions that globally alter glucose homoeostasis.

#### Receptor-specific agents

The high-affinity binding of IGFs to IR-A and IGF1R offers potentially useful pharmacological targets, and antibodies to IGF1R and IR-A, as well as various tyrosine kinase inhibitors (TKIs), have been tested in early phase clinical trials.^45^ However, given the ubiquitous nature of both IR and IGF1R in human tissues, the toxicities associated with targeting these receptors pose serious challenges. Furthermore, strategic targeting has proven to be difficult. On the one hand, blocking both IGF1R and IR can result in dose-limiting hyperglycaemia,^46,47^ but, on the other hand, exclusively inhibiting IGF1R can cause compensatory activation of IR signalling.^48,49^ Investigation of various targets are ongoing, but for the purposes of this review, we will highlight some that are the most advanced in clinical development.

Figitumumab, an IGF1R monoclonal antibody, was investigated in Phase 3 clinical trials in combination with carboplatin and paclitaxel for the treatment of advanced non-small-cell lung cancer (NSCLC). However, the trial was closed early due to an increased incidence of serious adverse events, including grade 3/4 hyperglycaemia and treatment-related deaths.^50^ Two other IGF1R monoclonal antibodies, ganitumab and dalotuzumab, were investigated for the treatment of metastatic pancreatic cancer and metastatic colon cancer, respectively, but both trials were also terminated after preplanned futility assessments.^51,52^ In oestrogen receptor-positive (ER^+^) breast cancer, ganitumab in combination with the aromatase inhibitor exemestane or the ER downregulator fulvestrant failed to improve PFS and also induced significant rates of grade 3/4 hyperglycaemia.^53^ Other IGF1R monoclonal antibodies, including cixutumumab, robatumumab and istiratumab, have been investigated in Phase 1 and Phase 2 clinical trials but have shown limited efficacy and poor tolerability.^46,54–56^

Small-molecule TKIs targeting IGF1R, IR-A and IR-B have also been studied in the clinical setting. Although dual targeting of IGF1R and IR circumvents compensatory IR activation, this approach leads to a higher rate of hyperinsulinaemia and hyperglycaemia.^57^ In a Phase 3 randomised controlled trial (RCT), no difference in overall survival was seen in patients with adrenocortical carcinoma treated with linsitinib, which targets IGF1R and IR, versus those receiving placebo.^58^ The combination of linsitinib and paclitaxel chemotherapy did not improve survival in ovarian cancer, and linsitinib maintenance with erlotinib, a TKI of the epidermal growth factor receptor (EGFR), did not improve overall survival in patients with NSCLC.^59,60^

#### Ligand-specific agents

As well as inhibiting IGF1R and IR, other potential strategies include targeting the IGFs. Dusigitumab, a monoclonal antibody that binds IGF-2, has been explored in a Phase 1 basket trial of advanced solid malignancies and resulted in stable disease at best response, with a favourable toxicity profile.^61^ However, no further development of this agent is currently being planned. Early phase studies of xentuzumab, a monoclonal antibody that binds IGF-1 and IGF-2, have demonstrated promising anti-tumour activity in patients with breast cancer. No improvement in the overall PFS was reported with the addition of xentuzumab to exemestane and the mammalian target of rapamycin (mTOR) inhibitor everolimus. However, in patients without visceral metastasis, the three-drug regimen had a longer PFS (hazard ratio (HR): 0.21 (0.05–0.98)) compared with the combination of exemestane and everolimus alone.^62^

#### Agents that alter glucose homoeostasis

Repurposing medications labelled for the treatment of diabetes is an area of active investigation in cancer therapy. In preclinical models, metformin was shown to downregulate IGF signalling and inhibit proliferation of uterine serous carcinoma cells.^63^ Metformin also attenuates the expression of IGF1 and the activation of mTOR and Akt (downstream effectors of insulin signalling) in breast, lung and pancreatic cancer cells.^64–66^ However, the clinical response to metformin has been mixed. When combined with other cytotoxic agents during neoadjuvant treatment of breast cancer, metformin improved pathological complete response rates, but did not improve PFS in the metastatic setting.^67,68^ Similarly, the data supporting metformin in the treatment of endometrial cancer have been mixed. Inhibiting the IGF1 and PI3K signalling pathways with metformin lowers cellular proliferation in endometrial tumours.^69,70^ In small window-of-opportunity (presurgery) trials, metformin reduced tumour proliferation (as indicated by the marker Ki-67) by 11.75% (*P* = 0.008) in patients in one trial and 17.2% (*P* = 0.002) in another trial, but these findings were not replicated in a confirmatory Phase 3 trial.^71–73^

Based on encouraging observational, preclinical and early phase data, several clinical trials testing metformin in the presurgical/neoadjuvant, adjuvant and metastatic settings in combination with standard anti-tumour therapies are ongoing.

## Obesity and dysregulated adipokine signalling

Dysregulated circulating levels of adipokines—hormones and cytokines secreted by adipose tissue—is a hallmark of hyperadiposity and can promote tumour growth. The primary function of one such adipocyte-secreted hormone and biomarker of adiposity,^74,75^ leptin, is hypothalamic-mediated regulation of appetite, which modulates feeding behaviour and energy expenditure.^76,77^ Circulating levels of leptin are elevated in obese individuals and are associated with an increased risk of the development and progression of cancer, such as endometrial, breast, colon, and kidney cancers, among others.^78–80^

**Fig. 2:**
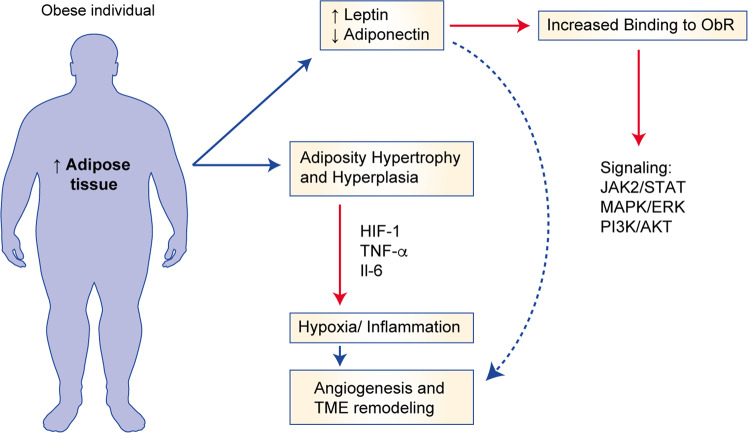
Effects of obesity on adipokine signalling. ObR leptin binding receptor, HIF-1 hypoxia-inducible factor, TNF-alpha tumor necrosis factor alpha, IL-6 interleukin 6, TME tumor microenvironment.

The mechanisms through which leptin promotes cancer growth are best outlined in the setting of breast cancer. Binding of leptin to one of the six isoforms of the leptin receptor induces the activation of various signalling pathways including the Janus kinase/signal transducer and activator of transcription (JAK/STAT), MAPK and PI3K pathways, which ultimately promote cell proliferation.^81,82^ Leptin signalling through the leptin receptor also activates mammary cancer stem cells and is necessary for mammary stem cell survival and maintenance^83^ (Fig. 2). Insulin and IGF1 can also increase the expression of leptin and its receptor in mammary epithelial tissues, and this increased expression is associated with worse prognosis in breast cancer.^79^ Furthermore, the mRNA and protein expression of leptin in breast cancer cells can be regulated by hyperinsulinaemia and hypoxia (through hypoxia-inducible factor (HIF)).^84^ In turn, leptin can stimulate angiogenesis and create vascular permeability to enable further malignant cell growth.^85,86^ Leptin is, therefore, an important mediator of interactions between the tumour and the tumour microenvironment (TME).

Another adipocyte-secreted hormone is adiponectin, which has anti-diabetic and anti-inflammatory properties. Plasma levels of adiponectin are decreased in obesity and metabolic syndrome, as is the expression of adiponectin receptors (AdipoR1 and AdipoR2), resulting in further reduced adiponectin sensitivity.^87^ Low adiponectin levels are associated with insulin resistance and an increased risk of obesity-associated malignancies, including breast and endometrial cancer.^88–91^ The mechanisms contributing to this relationship are not known; however, adiponectin has been shown to inhibit the growth of several cancer cell lines in vitro.^92^ Additionally, adiponectin activates the 5’-adenosine monophosphate-activated protein kinase (AMPK) pathway, leading to upregulation of p53 and p21, key regulators of the cell cycle and apoptosis.^93^ Furthermore, because adiposity increases leptin and decreases adiponectin levels, the leptin/adiponectin ratio has been suggested to be a predictor of breast cancer growth.^94,95^ The levels of other adipokines, such as resistin and visfatin, are elevated in obesity; these increased levels are markers of inflammation, and are associated with the development and progression of various cancers.^96–99^ For example, resistin is thought to promote growth of breast cancer cells through Toll-like receptor 4 (TLR4)-mediated activation of nuclear factor (NF)-κB and STAT3.^96^

### Targeting adipokine signalling and repurposing diabetic and cardiometabolic medications

#### Diabetic agents that inhibit leptin signalling

Despite the well-established role of leptin in promoting tumour growth, no pharmacological interventions directly targeting leptin signalling are currently approved for the prevention or treatment of cancer. Interestingly, metformin has been shown to decrease leptin levels in patients with either breast or endometrial cancer.^100,101^ In patients with endometrial cancer, metformin reduces cancer cell proliferation (as measured by Ki-67 staining) and has inhibitory effects on the PI3K–mTOR signalling pathways in the presurgical window.^71,72^ In a trial of 200 non-diabetic patients with breast cancer, metformin did not significantly decrease breast cancer cell proliferation. However, trends were identified in an unplanned analysis of Ki-67 reduction in overweight women with insulin resistance.^102^ Metformin is currently being tested for adjuvant breast cancer treatment in the MA.32 trial, a Phase 3 multicentre trial that has completed accrual with results anticipated after maturation of follow-up data.^103^ A leptin receptor antagonist has been investigated in preclinical models in triple-negative breast cancer.^104,105^ Pegylated leptin peptide receptor antagonist 2 (PEG-LPrA2) was shown to inhibit leptin signalling pathways and inhibit breast cancer growth both in vitro and in vivo in breast cancer xenograft models.^106^ These promising preclinical findings warrant further investigation in early phase human trials.

#### Diabetic agents that increase adiponectin levels

Peroxisome-proliferator-activated receptor γ (PPARγ) synthetic ligands, such as rosiglitazone and pioglitazone, are diabetes drugs that regulate glucose metabolism, reduce hyperinsulinaemia and alter fatty acid metabolism.^107^ Additionally, PPARγ synthetic ligands have been shown to increase adiponectin levels in preclinical models and in humans.^108–112^ Based on the observations that low adiponectin levels are associated with cancer progression as discussed above, the propensity of PPARγ agonists to increase adiponectin levels may be beneficial for treating obesity-driven cancers.^113,114^

#### Statins

Statins, which are widely used for the management of lipid levels, might also have anticancer properties, and many preclinical studies have suggested a protective role for statins against cancer development and progression.^115–120^ Several mechanisms have been proposed to underlie this anticancer effect: impaired tumour-cell proliferation via inhibition of Ras and Rho activation;^121,122^ inhibition of cellular proliferation via cell cycle arrest;^123^ induction of apoptosis;^116,124,125^ dose-dependent inhibition of angiogenesis;^126^ and anti-inflammatory properties.^118–120^ Epidemiology data supporting an anticancer effect of statins have been mixed. Several population studies have reported a reduced risk of breast cancer in statin users compared with non-statin users,^127–131^ but meta-analyses have not confirmed this association.^132–136^ Notably, high-dose statin consumption might provide a greater anticancer effect.^130^ Additionally, whether the statin is hydrophobic or lipophilic could differentially affect cancer risk, although data on this point are conflicting.^128,131^ The use of statins might also be associated with reduced cancer mortality after diagnosis,^137^ although observational studies do not support this link in breast cancer.^138–140^ Taken together, the epidemiology reports to date provide a signal that statin use might be protective against breast cancer for some, but not all, patients. Identifying this high-risk or statin-responsive population will be critical to developing successful intervention and prevention strategies that use statins.

## Oestrogen signalling

**Fig. 3:**
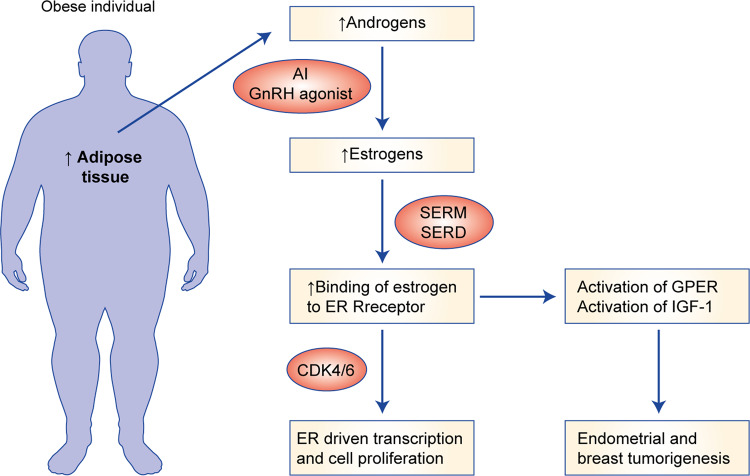
Effects of obesity on oestrogen signalling. AI aromatase inhibitor, GnRH gonadotropin releasing hormone, SERM selective estrogen receptor modulator (i.e; tamoxifen), SERDselectve receptor degrader (i.e.; fulvestrant), CDK4/6 cyclin dependent kinases 4 and 6 inhibitors, GPER G-coupled estrogen receptor.

It has long been established that oestrogen signalling is a key driver of various cellular processes including cell proliferation and survival, and that removal of the source of oestrogens—predominantly the ovaries in premenopausal women—provides clinical benefit and tumour regression for oestrogen-sensitive cancers.^141^ Increased levels of oestrogen function to increase cell proliferation and angiogenesis through various mechanisms,^142^ including binding to the ER and stimulating the IGF1 signalling pathway in breast cancer;^143^ in endometrial cancer, oestrogen binding to the G-protein-coupled oestrogen receptor (GPER) can result in hyperplasia in endometrial tissue.^144^ Furthermore, through activation of GPER, oestrogens play a role in hypoxia-induced angiogenesis in breast cancer^145^ (Fig. 3).

After menopause, the main source of systemic oestrogen comes from the peripheral conversion of androgens by the oestrogen biosynthetic enzyme, aromatase, and one of the most well-characterised obesity-related mechanisms for cancer pathogenesis involves the increased activity of aromatase in adipose tissue, consistent with the dysregulation of oestrogens being implicated in the development of obesity-associated ER^**+**^ breast and endometrial cancers.^11,146,147^ Obesity and metabolic syndrome have been linked to increased inflammation and increased expression in breast tissue and adipose stromal cells of the aromatase-encoding gene *CYP19A1.*^148,149^ In vitro studies using isolated primary human breast preadipocytes or adipose stromal cells, the main cell type responsible for oestrogen biosynthesis in the breast, have contributed to defining the mechanism by which inflammatory mediators drive aromatase expression in the context of obesity. For example, prostaglandin E2 (PGE_2_), a crucial inflammatory mediator, has been shown to strongly stimulate the expression of *CYP19A1* via activation of PII, the promoter contributing to the majority of aromatase transcripts in breast tissue in both obesity and breast cancer.^150–152^ This increased expression is dependent on the binding and activity of a number of transcription factors and co-regulators,^153–161^ and is regulated by several pathways,^152,159,160,162–164^ some of which, notably, involve regulation by leptin.^148,159^ Conversely, p53 has been shown to act as a transcriptional repressor of the *CYP19A1* gene, but is inhibited by both PGE_2_ and leptin.^148,165^ The effects of p53 in tumour suppression therefore go beyond its established role in promoting cell cycle arrest and apoptosis.^166^ Other inflammatory mediators, such as tumour necrosis factor (TNF) and interleukin (IL)-6, have also been shown to stimulate the expression of the *CYP19A1* promoter I.4.^167–170^

### Targeting oestrogen signalling

#### Aromatase inhibitors

Metformin has been identified as a potential breast-specific aromatase inhibitor.^171,172^ Interestingly, the adipokine adiponectin and the hunger hormone ghrelin have also been shown to suppress aromatase expression in a promoter-specific manner, which may help to explain the association between low levels of adiponectin and breast cancer growth in the setting of obesity.^159,173,174^ It remains to be determined whether these results can be leveraged to improve treatment of obesity-related breast cancer.

Specific steroidal and non-steroidal aromatase inhibitors have demonstrated efficacy for the prevention and treatment of ER^+^ breast cancers, with an approximate 50% reduction in the risk of ER^+^ breast cancer development or recurrence.^175^ Aromatase inhibitors might also have clinical utility in ER^+^ endometrial cancers, although the efficacy of aromatase inhibitors for the treatment of endometrial cancer is modest.^176^ Anastrozole, a non-steroidal aromatase inhibitor, has been shown to reduce proliferation in endometrial cancer cells when used in the neoadjuvant setting, and has modest activity for the treatment of recurrent ER^+^ endometrial cancer.^177,178^ Letrozole, another non-steroidal aromatase inhibitor, in combination with everolimus, an mTOR inhibitor, is associated with an overall response rate of 32% in an unselected endometrial cancer population.^179^ Exemestane, an irreversible steroidal aromatase inhibitor, is currently being tested for the treatment of endometrial hyperplasia and low-grade endometrial cancer (NCT03300557). Other ongoing trials are assessing various combinations of aromatase inhibitors with inhibitors of the PI3K–mTOR pathway (NCT02730923, NCT03008408). As well as the use of aromatase inhibitors, targeting the ER is a promising strategy for the treatment of ER^+^ endometrial cancer. An ongoing clinical trial is assessing fulvestrant, a selective ER downregulator, in combination with abemaciclib, a cyclin-dependent kinase (CDK)4/6 inhibitor, for the treatment of ER^+^ endometrial cancer (NCT03643510). Whether obesity affects the efficacy of various hormone therapies for endometrial cancer is currently unknown and warrants further investigation. In the setting of breast cancer, however, obesity is associated with reduced efficacy of aromatase inhibitors.^180^ This observation may be explained in part by increased expression of aromatase in the breast due to obesity-related adipose tissue inflammation, which will be discussed below.

## Obesity and the microenvironment

The tumour microenvironment has an established role in tumour formation and metastatic invasion. It consists of various cells including lymphocytes, antigen presenting cells, cancer fibroblasts and the extracellular matrix. Increased adiposity can create chronic inflammation and hypoxic conditions that disrupt the intricate web of connections, and subsequent perturbations contribute to carcinogenesis.

### Changes in vascularity

In the context of a tumour, it is well established that the increasing mass resulting from rapidly dividing cells generates hypoxic areas; HIF-1α mediates the adaptive response to the low availability of oxygen, with higher levels of HIF-1α promoting angiogenesis, thereby supporting further tumour growth and metastasis^181,182^ (Fig. 1). Indeed, higher levels of HIF-1α have been associated with recurrence, metastasis and reduced survival in several tumour types.^183,184^ The mechanisms underlying these observations and potential opportunities to intervene have been reviewed elsewhere by Pouysségur and colleagues.^185^ Hypoxia also induces the expression of vascular endothelial growth factor (VEGF), which further promotes angiogenesis and tumour growth.^185^ Obesity is also associated with an increase in tissue hypoxia due to expansion of adipose tissue beyond its vascular supply,^186^ which also promotes neovascularisation.^187^ Furthermore, hypoxia-induced VEGF expression can promote adipose tissue expansion, as well as inflammation, and this can generate a microenvironment that is supportive of tumour growth (discussed below).^186,188^ In endometrial cancer, VEGF is upregulated in the visceral adipose tissue of obese women and drives endometrial hyperplasia and endometrial cell growth through the PI3K–Akt–mTOR pathway.^189^ Anti-VEGF therapies used to target breast cancer have failed to improve overall survival, and preclinical evidence suggests that this might be related to obesity-induced resistance to anti-VEGF therapy by the production of inflammatory factors such as IL-6, which, as alluded to above, can promote a favourable TME.^190^ Small retrospective studies in ovarian and colorectal cancer have suggested that increased adiposity is associated with decreased efficacy of bevacizumab.^191,192^

#### Targeting angiogenesis

Inhibitors of HIF are currently under investigation for the treatment of various types of cancer in early phase clinical trials. For example, Phase 1 trials of EZN-2968 (a HIF-1 inhibitor) and PT2977 (a HIF-2 inhibitor) demonstrated some clinical activity, suggested by prolonged stable disease (>24 weeks) in one patient with a duodenal neuroendocrine tumour and five responses (one partial response, four stable disease) in six patients with clear cell renal cell carcinoma.^193,194^ Inhibitors of VEGF signalling have progressed further than HIF inhibitors in clinical development. The anti-VEGF monoclonal antibody bevacizumab is currently used for the treatment of lung, colon, cervical and ovarian cancers,^195–198^ while ramucirumab, an anti-VEGF receptor antibody, is also approved for the treatment of gastric, colorectal and hepatocellular cancers.^199–201^ Finally, small-molecule TKIs of VEGF signalling, such as sorafenib, sunitinib, pazopanib, lenvatinib and others, have demonstrated efficacy for the treatment of kidney, thyroid and hepatocellular cancers.^202–204^ In endometrial cancer, lenvatinib in combination with the checkpoint inhibitor pembrolizumab has FDA breakthrough designation and is undergoing confirmatory Phase 3 investigation.^205^

### Chronic inflammation

Obesity is associated with a chronic state of subclinical inflammation that is characterised by white adipose tissue inflammation. Such inflammation can be histologically detected by the presence of crown-like structures (CLS),^206^ in which dead or dying adipocytes are surrounded by activated macrophages. These macrophages are associated with the production of several pro-inflammatory mediators, the expression of aromatase, and the presence of a fibrotic extracellular matrix.^207,208^ In humans, adipose inflammation in the breast is present in many overweight/obese individuals and is associated with postmenopausal status.^11^ In preclinical models of postmenopausal obesity, inflammation of mammary adipose tissue is associated with increased levels of TNF-α, IL-1β, IL-6 and cyclo-oxygenase (COX)-2 and an increased risk of developing breast cancer and reduced distant disease-free survival after breast cancer diagnosis.^209,210^ These chronic inflammatory changes associated with dysfunctional adipose tissue contribute to a microenvironment that is rich in tumour growth factors.^211,212^ We have previously reviewed the mechanisms through which this pro-inflammatory microenvironment promotes tumour growth.^213^ Interventions that reduce adipose inflammation, such as diet and exercise, might therefore reduce breast cancer risk and/or mortality, and clinical trials investigating the effects of diet and exercise on cancer-related outcomes are currently underway.

## Non-pharmacological/lifestyle interventions

We have so far outlined the various mechanisms through which increased adiposity drives changes in insulin signalling, adipokine signalling, oestrogen signalling and in the TME, including angiogenesis and chronic inflammation. We have also briefly addressed the current landscape of pharmacological interventions in the context of cancer treatment for targets that are dysregulated by obesity. However, as well as such targeted approaches, the pleiotropic effects of lifestyle interventions offer a promising strategy to reverse the cancer-promoting effects of obesity. Furthermore, combining lifestyle interventions with pharmacological therapies could further augment the efficacy of anticancer therapies.

### Dietary interventions

Although the biological mechanisms through which the modulation of specific macro- and micro-nutrients impact tumour biology are beyond the scope of this discussion and have been reviewed elsewhere,^214,215^ we outline here the key findings from RCTs that have tested strategies to shift overall dietary patterns in cancer populations. In the case of breast cancer, several trials have established that dietary modification as well as exercise are achievable and safe after diagnosis.^216,217^ Subsequent trials have examined the effects of diet and exercise interventions on weight loss, breast cancer outcomes, and circulating blood factors (Table 1). Two large RCTs that tested dietary interventions to improve breast cancer outcomes have been completed, but the results are conflicting. The Women’s Intervention Nutrition Study (WINS) demonstrated a 24% reduction in the recurrence of breast cancer in patients randomly assigned to a low-fat diet group versus control patients.^218^ Conversely, however, the Women’s Healthy Eating and Living (WHEL) trial did not show any improvement in the risk of recurrence for women randomised to a low-fat, high-fibre diet;^219^ diets high in fibre are known to increase microbial biodiversity (see below) and decrease insulin resistance.^220,221^ The long-term results of another RCT, the Women’s Health Initiative (WHI), were reported in 2019 and demonstrated a 21% reduction in mortality after breast cancer diagnosis in patients randomised to a low-fat diet intervention compared with a usual diet.^222^ Although large-scale clinical trial data are still lacking in this area, several RCTs testing the efficacy of diet and/or exercise interventions are ongoing (Table 2).

**Table 1:** Completed lifestyle randomised control trials (RCT) for cancer survivors.

Study	Population	Intervention	BMI	Primary endpoint	Outcomes
*Breast cancer trials*					
WINS	Early stage BC	Fat reduction diet	All	RFS	9.8% vs 12.4% (HR 0.78; CI 0.60–0.98) *P* = 0.03^156^
WHEL	Early stage BC	Diet	All	Recurrence rate death	16.7% vs 16.9% (HR 0.96; CI 0.8–1.17) *P* = 0.63 10.1% vs 10.3% (HR 0.91; CI 0.72–1.15) *P* = 0.43^157^
DAMES	Mother-Daughter Dyads with early Stage BC	Diet + PA	25–39.9	Feasibility & weight loss	>5% weight loss in 21.7-39.1% of participants^167^
LISA	Node negative BC	Diet + PA	24–50	DFS events*	12.9% vs 18.0% (HR 0.71; CI 0.41–1.24) *P* = 0.23^181^
ENERGY	Early stage BC	Diet + supervised exercise	25–45	Weight loss	3.7% vs 1.3% at 24 months (*P* < 0.001)^168^
LEAN	Survivors of stage 0-III BC	Diet + PA	≥25	Weight loss	6.4% vs 5.4% vs 2.0%** (*P* = 0.004, *P* = 0.009, *P* = 0.46)^169^
SUCCESS C	Her2-negative early stage BC	Diet + PA	24–40	DFS	No difference in DFS. HR 0.99; CI 0.76–1.28, *P* = 0.922^182^
*Prostate cancer trials*					
MEAL	Localized PC	Diet	All	Time to progression	No difference detected. Adjusted HR 0.97 (CI 0.76–1.25), *P* = 0.84)^183^
CAPS2	Localized PC	Diet	≥24	PSADT***	28 vs 13 months, *P* = 0.021^184^
*Endometrial cancer trials*					
SUCCEED	Stage I-II EC	Diet + PA	≥25	Weight loss	1.4 kg vs −4.6 kg (CI −1.09 to 0.14), *P* = 0.011^185^
*Multiple cancer trials*					
RENEW	Survivors of BC, CRC, PC	Diet + PA	25–40	PF scale decline	−2.15 vs −4.84, *P* = 0.03^186^

*PA* physical activity, *EC* endometrial cancer, *BC* breast cancer, *PF* physical function, *CRC* colorectal cancer, *PC* prostate cancer, *I* individual arm, *T* team arm, *PSADT* prostate serum antigen doubling time, *CC* colon cancer, *HR* hazard ratio, *CI* confidence interval.

*Loss of funding, underpowered, reporting weight loss, **in-person vs telephone vs standard care, ***study terminated after interim analysis showed futility.

**Table 2:** Ongoing lifestyle randomised control trials (RCT) in cancer patients.

Study	Population	Intervention	BMI	Primary endpoint
*Breast cancer*				
DIANA-5^170^	Early stage BC	Diet + exercise	All	Recurrence
PREDICOP (NCT02035631)	Early stage BC	Diet + supervised exercise	18–40	Time to recurrence
BWEL (NCT02750826)	Her2-negative early stage BC	Diet + PA	≥27	Invasive DFS
DEDiCa (NCT02786875)	Early stage BC	Diet	All	DFS
DIRECT (NCT02126449)	Stage II/II Her2-negative BC	Diet	≥19	Toxicity
Efficacy of Dietary Fat Reduction (NCT00002564)	Stage I/II/IIIA BC	Diet	All	DFS, OS
OPTITRAIN (NCT02522260)	Early stage BC	Exercise	All	Cancer-related fatigue
EXCAP (NCT00851812)	Early stage BC	Exercise	All	Cancer-related fatigue
*Colon cancer*				
CHALLENGE^187^	Stage II/III CRC	Exercise	All	DFS
*Prostate cancer*				
INTERVAL (NCT02730338)	MCRPC	Exercise	All	OS
*Endometrial cancer*				
REWARD (NCT01870947)	Stage I EC	Exercise	≥30.0	Weight change
Step into Wellness (NCT03367923)	Stage IA–IIIA EC	Exercise	25–60	Activity level
*Ovarian cancer*				
LIVES (NCT00719303)	Stage II–IV OC	Diet + PA	>20	PFS

*PA* physical activity, *RFS* relapse-free survival, *PF* physical functioning, *OS* overall survival, *DFS* disease-free survival, *EC* endometrial cancer, *OC* ovarian cancer, *PFS* progression-free survival, *MCRPC* metastatic castrate-resistant prostate cancer, *CRC* colorectal cancer.

For patients diagnosed with endometrial or breast cancer, preclinical evidence suggests that a ketogenic diet (KD; a diet of high fats, moderate proteins, and very low carbohydrates) might improve the efficacy of PI3K inhibitors by inhibiting insulin signalling.^44,223^ It has recently been shown in murine KPC tumour models that treatment with PI3K inhibitors causes a transient hyperglycaemia and hyperinsulinaemia. This resultant hyperinsulinaemia can partially reactivate PI3K signalling, and following PI3K inhibition, can reactivate PI3K signalling in both normal and tumour tissues.^44^ A ketogenic diet, which is deficient in carbohydrate, prevents hyperinsulinaemia and can thereby reduce the paradoxical reactivation of PI3K by PI3K inhibitor-associated hyperglycaemia.^44^

RCTs of a KD have demonstrated reductions in visceral adiposity and serum insulin levels without adversely affecting blood lipid levels despite elevated dietary fat intake.^224,225^ Notably, in xenograft models of pancreatic cancer, a KD also increased sensitivity to radiation—putatively by reducing oxidative stress; however, the diet was poorly tolerated in a pilot study of nine people.^226^

In obese patients without malignancy, a very-low-calorie KD reduces visceral adiposity and obesity-related metabolic dysfunction, restores leptin and resistin levels to normal, and reduces the expression of inflammatory markers.^227–229^ This approach might therefore be particularly valuable for cancer populations where weight loss is a critical priority. The definition of a KD varies among clinical trials—all KDs include low carbohydrates, but varying cut-offs for daily calories and lipid targets exist.^230^ Thus, the tolerability and durability of a KD intervention requires further testing, which would be aided by standardisation of KD parameters. Finally, it is important to note that, although a KD might be beneficial for certain established tumour phenotypes (e.g., *PIK3CA*-mutated tumours), this approach might not be effective—and could potentially be detrimenta—in certain other tumour types and in the preventive setting. For example, high dietary fat intake has been associated with an increased risk of developing breast cancer.^231,232^ Accordingly, the selection of an appropriate KD protocol (e.g., low-calorie, carbohydrate-restricted, and/or limited-fat) will be important for the development of this approach for use in cancer populations. However, it is important to note that a KD might not be beneficial in all circumstances or cancer histologies, as other groups have noted that changing to a high protein intake can increase insulin signalling through IGF-1.^220,221^ There remains ambiguity regarding which diets can effectively reverse tumorigenesis mechanisms, and future studies should aim to identify the appropriate populations and tumour phenotypes for rational dietary intervention.

#### The gut microbiome

Investigations carried out over the past decade have demonstrated that particular gut microbiome signatures are associated with the development of cancer,^233,234^ and that alterations in the gut microbiota can promote chronic inflammation and immunological changes that facilitate carcinogenesis.^235,236^ As obesity and diet alter the health and diversity of the gut microbiome, research on the role of the gut microbiome in contributing to obesity-associated cancers is active and ongoing. Although there is less data regarding hormonally driven cancers such as breast and endometrial cancers, an individual’s metabolic profile and oestrogen status can affect their microbiome. In breast cancer, there is a growing interest in the ‘oestrobolome’, which includes genes that encode bacterial enzymes such as β-glucuronidases, which are involved in the processing of endogenous oestrogens,^237^ and understanding how changes in oestrogen-dependent pathways influence the gut microbiome.^238^ Obesity can disrupt this oestrobolome, resulting in increased levels of oestrogen and its metabolites, which could affect the development and treatment of breast and endometrial cancers.^239,240^

### Exercise interventions

A substantial body of observational data suggests that post-diagnosis exercise could prevent cancer progression and improve cancer-related mortality. In a seminal study by Holmes et al.,^241^ 9–14.9 MET (metabolic equivalent of task) hours·per week (equivalent to ~150–250 min of moderate-intensity exercise per week) was associated with an adjusted 50% reduction in breast cancer death compared with <3 MET-hours per.week among 2987 patients with primary breast cancer. In another systematic review, post-diagnosis exercise was associated with, on average, a 37% reduction (95% confidence interval (CI) 0.54–0.73) in the risk of cancer-specific mortality in the most- versus least-active patients.^242^ Collectively, observational data support the hypothesis that exercise confers anti-tumour effects for several cancer types.

Although data from investigations into the effect of post-diagnosis exercise on cancer progression from prospective RCTs are not yet available, such trials are underway and outlined in Table 2. The Colon Health and Life-Long Exercise Change (CHALLENGE) trial is an international, multicentre, Phase 3 trial investigating the impact of exercise on recurrence and cancer-specific mortality in patients with resected high-risk stage II or stage III colorectal cancer.^243^ Another international, multicentre Phase 3 trial, the INTense Exercise foR survival (INTERVAL) trial, is investigating the effects of high-intensity aerobic and resistance training on disease outcomes in 866 patients with metastatic castrate-resistant prostate cancer (NCT02730338). Data from these, and other, Phase 3 trials of exercise in cancer populations are eagerly awaited, but it is important to note that the ‘dose’ of exercise that confers optimal anticancer efficacy or predictors of favourable response to exercise has not yet been identified. Early phase dose-finding trials of exercise are needed, and a Phase 1a/1b trial of exercise in ER^+^ metastatic breast cancer is currently ongoing (NCT03988595).

### Combination diet and exercise interventions

Several RCTs have demonstrated that combining diet and exercise interventions provides an effective approach for inducing weight loss in patients who have survived obesity-related breast cancer^244–246^ (Table 1). Several of these weight loss interventions have also demonstrated improvements in circulating metabolic and inflammatory factors.^246–250^ For example, in the Lifestyle, Exercise, and Nutrition (LEAN) study, breast cancer survivors with a BMI ≥25 randomly assigned to diet and physical activity counselling experienced reductions in the level of circulating C-reactive protein (CRP) and body fat percentage compared with usual care.^246^ Participants who achieved a 5% or greater weight loss by caloric restriction and increased physical activity were also found to have reductions in their levels of circulating insulin, leptin and IL-6.^246^ Several other studies have established that weight loss is an effective method for reducing circulating levels of CRP, insulin, glucose and lipids.^251–255^

Diet and exercise interventions can also influence the levels of circulating hormones in individuals with or without malignancy. In the Nutrition and Exercise for Women (NEW) trial, circulating levels of estrone and oestradiol in overweight and obese postmenopausal women were reduced with energy-restricted diet, exercise, or combined diet plus exercise, versus control.^256^ The interventions also increased the circulating levels of sex-hormone-binding globulin (SHBG) and decreased free oestradiol and testosterone levels, which could inhibit the recurrence or growth of hormone-sensitive tumours. The magnitude of effect on SHBG and oestrogens was greatest in the diet plus exercise arm. Encouraging findings from these trials collectively support the further development of diet and exercise interventions in the prevention and treatment of cancer.^257^

### Bariatric surgery

Given the various mechanisms by which obesity contributes to carcinogenesis, weight loss mediated by bariatric surgery has been investigated as a strategy for adjunct cancer treatment and prevention. The Swedish Obesity Study demonstrated that, especially for women, bariatric surgery reduced the incidence of cancer with a HR of 0.67 (95% CI 0.53–0.85);^258^ this risk reduction was confirmed in a large multicentre retrospective study in the USA in obesity-related cancers including breast cancer (HR 0.58; 95% CI 0.44–0.77) and in endometrial cancer (HR 0.50; 95% CI 0.37–0.67).^259^ A prospective trial is investigating the efficacy of bariatric surgery in reducing recurrence in breast cancer patients (NCT03946423).^260^ Although additional randomised prospective data are needed, it seems that weight loss modulates many of the effects of obesity on carcinogenesis.

## Future directions

**Fig. 4:**
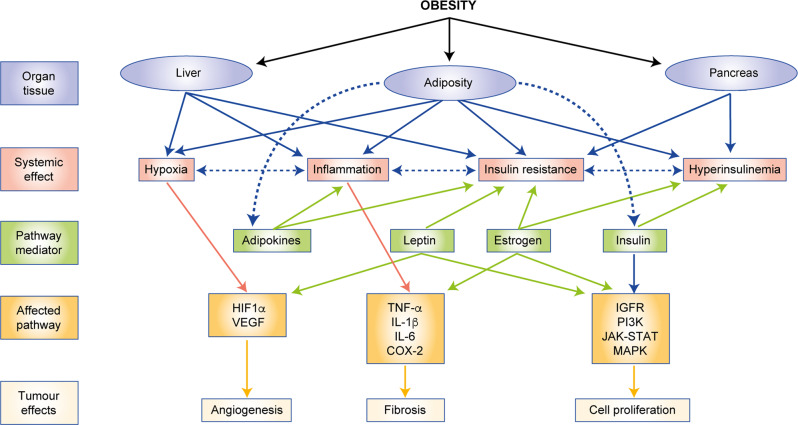
Summary of mechanisms through which obesity promotes tumorigenesis. HIF1α hypoxia- inducible factor 1- alpha, VEGF vascular endothelial growth factor, TNFα tumor necrosis factor alpha, IL-1β interleukin 1 beta, IL- 6 interleukin 6, COX-2 cyclooxygenase isoenzyme 2, IGFR- insuline-like growth factor receptor, JAK-STAT Janus kinases-signal transducer and activtor of transcription proteins, MAPK mitogen-activated protein kinase.

As increasing data elucidate the mechanisms by which obesity can alter cancer cell signalling, the prospective TME and systemic factors, additional targets that can be therapeutically exploited to improve obesity-related cancer risk and outcomes are likely to be identified. Figure 4 provides a summary of the pathways and mechanisms through which obesity promotes tumour growth, which establishes the paradigm for interventions. A number of pharmacologic agents could be repurposed for the prevention and treatment of obesity-related cancers, and obesity might be associated with a differential response to existing and novel anticancer therapies. Lifestyle interventions, including dietary modification and exercise, also demonstrate potential anticancer efficacy; however, the identification of appropriate ‘dose’, populations and tumour phenotypes is needed to leverage the promise of this approach. Significant progress has been made in elucidating the mechanisms through which obesity promotes cancer risk and mortality. Interestingly, a number of pathways that are dysregulated in obesity are also key drivers of oestrogen production, cancer growth and angiogenesis. Targeting these pathways would therefore potentially lead to a multifaceted approach to tumour suppression through both direct and indirect mechanisms. Translating these findings into effective clinical strategies is urgently needed to halt the accelerating global burden of obesity-related cancer.

## Data Availability

Not applicable.
